# Patient Engagement in the Design of a Mobile Health App That Supports Enhanced Recovery Protocols for Cardiac Surgery: Development Study

**DOI:** 10.2196/26597

**Published:** 2021-11-30

**Authors:** Anna M Chudyk, Sandra Ragheb, David Kent, Todd A Duhamel, Carole Hyra, Mudra G Dave, Rakesh C Arora, Annette SH Schultz

**Affiliations:** 1 Department of Family Medicine Rady Faculty of Health Sciences University of Manitoba Winnipeg, MB Canada; 2 Max Rady College of Medicine Rady Faculty of Health Sciences University of Manitoba Winnipeg, MB Canada; 3 Faculty of Kinesiology and Recreation Management University of Manitoba Winnipeg, MB Canada; 4 Institute of Cardiovascular Sciences St Boniface Hospital Winnipeg, MB Canada; 5 Healthy Heart Patient and Caregiver Researcher Group Institute of Cardiovascular Sciences St Boniface Hospital Winnipeg, MB Canada; 6 Department of Surgery, Section of Cardiac Surgery Max Rady College of Medicine University of Manitoba Winnipeg, MB Canada; 7 College of Nursing Rady Faculty of Health Sciences University of Manitoba Winnipeg, MB Canada; 8 Health Services & Structural Determinants of Health Research St Boniface Research Centre Winnipeg, MB Canada

**Keywords:** cardiac surgery, perioperative care, enhanced recovery protocols, mobile app, smartphone app, mHealth, development, patient and public involvement, patient engagement in research

## Abstract

**Background:**

Despite the importance of their perspectives, end users (eg, patients, caregivers) are not typically engaged by academic researchers in the development of mobile health (mHealth) apps for perioperative cardiac surgery settings.

**Objective:**

The aim of this study was to describe a process for and the impact of patient engagement in the development of an mHealth app that supports patient and caregiver involvement with enhanced recovery protocols during the perioperative period of cardiac surgery.

**Methods:**

Engagement occurred at the level of consultation and took the form of an advisory panel. Patients who underwent cardiac surgery (2017-2018) at St. Boniface Hospital (Winnipeg, Manitoba) and their caregivers were approached for participation. A qualitative exploration determined the impact of patient engagement on the development (ie, design and content) of the mHealth app. This included a description of (1) the key messages generated by the advisory panel, (2) how key messages were incorporated into the development of the mHealth app, and (3) feedback from the developers of the mHealth app about the key messages generated by the advisory panel.

**Results:**

The advisory panel (N=10) generated 23 key messages to guide the development of the mHealth app. Key design-specific messages (n=7) centered around access, tracking, synchronization, and reminders. Key content-specific messages (n=16) centered around medical terms, professional roles, cardiac surgery procedures and recovery, educational videos, travel, nutrition, medications, resources, and physical activity. This information was directly incorporated into the design of the mHealth app as long as it was supported by the existing functionalities of the underlying platform. For example, the platform did not support the scheduling of reminders by users, identifying drug interactions, or synchronizing with other devices. The developers of the mHealth app noted that key messages resulted in the integration of a vast range and volume of information and resources instead of ones primarily focused on surgical information, content geared toward expectations management, and an expanded focus to include caregivers and other family members, so that these stakeholders may be directly included in the provision of information, allowing them to be better informed, prepare along with the patient, and be involved in recovery planning.

**Conclusions:**

Patient engagement may facilitate the development of a detail-oriented and patient-centered mHealth app whose design and content are driven by the lived experiences of end users.

## Introduction

Enhanced recovery protocols (ERPs) are evidence-based care pathways aimed at standardizing perioperative care. In offering a multimodal and interdisciplinary approach to care, these protocols have been proposed as a clinical strategy to effectively address complex and multisystem vulnerabilities [1,2], like those commonly present in older adults undergoing cardiac surgery [3,4]. Mobile health (mHealth) refers to medical and public health practice supported by mobile devices (eg, smartphones, tablets, patient monitoring devices) [5]. mHealth apps have the potential to enhance the utility of ERPs by increasing the effectiveness of information delivery and patients’ (and caregivers’) retention of information regarding their health care plan [6,7]. There is some evidence to support the feasibility of using mHealth during inpatient recovery of patients who had undergone cardiac surgery [8]. However, researchers’ efforts to develop mHealth for the perioperative cardiac surgery setting (and in general) are often limited by the lack of involvement of end users (such as patients and caregivers) in research activities [9].

Patients, caregivers, and other health service users may be involved in mHealth development studies as research participants or coresearchers, using participatory methods such as user-centered design, the participatory action research framework, and the Center for eHealth Research and Disease Management Roadmap [10]. Patient engagement (also commonly referred to as patient and public involvement, patient involvement, and stakeholder engagement) in research is a form of participatory action research that involves the “coproduction” of research with patients and caregivers. It has been defined as the formation of meaningful and active collaborations between researchers and patients (including informal caregivers) in research governance, priority setting, conduct, and knowledge translation [11]. Lack of attention to end users’ perspectives during the development phase is one of the competing explanations for the relatively low uptake of mHealth by patients [9]. Thus, an important step toward more widespread adoption of patients and caregivers as coproducers of mHealth research is one that facilitates a better understanding of processes for engaging patients and caregivers in mHealth development studies.

This study was set within the context of a Canadian clinical research hospital where our research group is involved in the development and implementation of ERPs for cardiac surgery. As part of this work, we initiated a project that developed an mHealth app and determined its effectiveness in improving knowledge delivery of patient education materials and patient adherence to ERPs during the perioperative period of cardiac surgery. A feasibility study of the mHealth app is currently under review. This study focuses on the patient engagement process employed to develop the mHealth app, which was guided by the Canadian Institutes of Health Research’s Patient Engagement Framework [11] and our scoping review of models and frameworks of patient engagement in health services research [12]. Given the novelty of engaging patients as coproducers of mHealth in academic research settings and among most of our team members, this study aimed to describe a process for and the impact of patient engagement on the development of an mHealth app that supports ERPs for cardiac surgery.

## Methods

### Ethical Approval and Consent

This study was set in an academic tertiary care center that performs cardiac surgery (St. Boniface Hospital, Winnipeg, Manitoba). Ethical approval for this study was obtained from the University of Manitoba Research Ethics Board as well as the Research Review Committee at St. Boniface Hospital. Patients and caregivers provided written informed consent and were compensated CAD $50 (CAD $1=US $0.80, for time and transportation) in addition to the cost of parking per meeting that they attended. The Guidance for Reporting Involvement of Patients and the Public long-form checklist guided the reporting of patient engagement in this paper [13].

### Overview of the mHealth App

**Figure 1:**
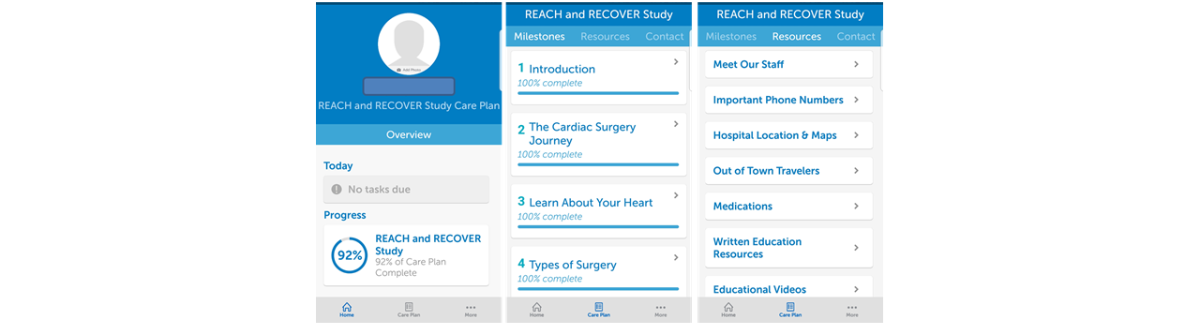
Screenshot of the mobile health app.

The mHealth under development was an app-based platform hosted by BeeWell Health [14]. This study gathered, adapted, and electronically formatted patient-and-caregiver derived content that addressed the patient journey from initial cardiac surgery consent through to the 8-week postoperative recovery period for delivery via the mHealth app. This content targeted 3 aspects of perioperative care (ie, patient-tailored education, optimization of patient health, and patient engagement in care) and focused on 4 domains of information (ie, nutrition, medications, resources, and physical activity). The 4 domains of information targeted by the mHealth app were informed by our previous work with patients who had undergone cardiac surgery and their caregivers (data unpublished). Specifically, focus group sessions identified these areas as priorities for patients who had undergone cardiac surgery and their caregivers. Continued research (ie, web-based and telephone surveys) validated these findings within a larger patient and caregiver population. A screenshot from the mHealth app is shown in Figure 1.

### Description of the Patient Engagement Process

**Figure 2:**
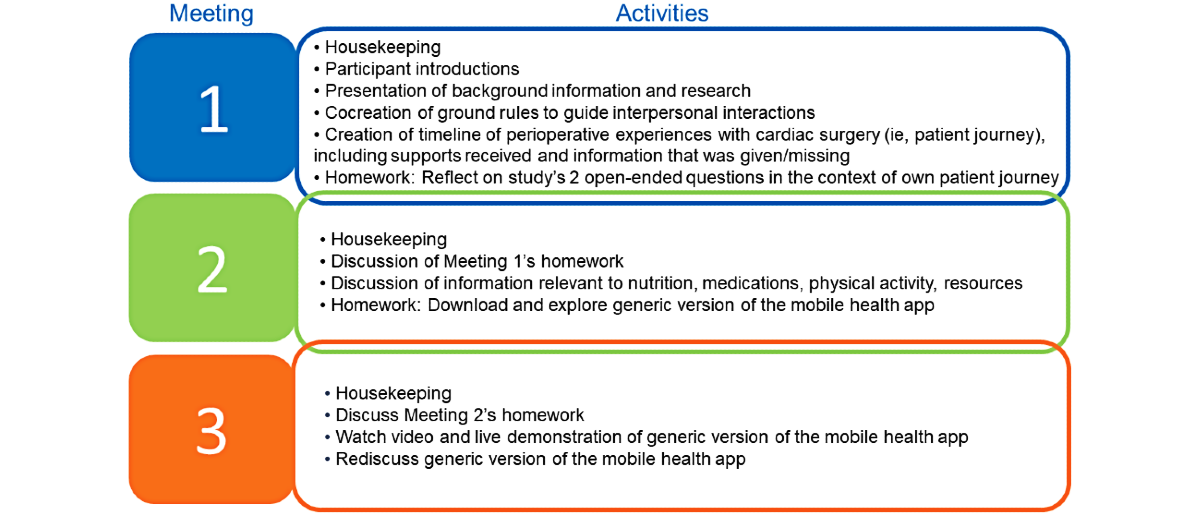
Outline of the activities in each meeting.

Patient engagement in research encompasses a wide range of activities and participation types, as influenced by the characteristics of a given project (eg, scope, time, financial resources) and the contributions patients are willing to offer [11,15-17]. In this study, engagement took the form of an advisory panel and occurred at the level of consultation [17]. The role of the advisory panel was to inform the development (operationalized as design and content) of the mHealth app. The advisory panel met in-person 3 times, approximately 2 weeks apart. Each meeting was approximately 3 hours in duration. Figure 2 displays an outline of the activities that occurred at each meeting. The activities that occurred within the meetings were not only developed to gather advisory panel input on the design and content of the mHealth app but also to create/facilitate an environment that supported the guiding principles that underlie patient engagement (ie, mutual respect, inclusiveness, cobuilding, support; see Multimedia Appendix 1 [11,18-20] for information on our approach to creating an environment that embodied these guiding principles). The primary method used to obtain advisory panel members’ input was group discussions. These discussions centered around 2 open-ended questions: “*what information stuck out as important during your patient journey*” and “*what information do you wish you had known during your patient journey.*” In addition, the scope of the discussions was narrowed to 4 domains of information (ie, nutrition, medications, resources, and physical activity) identified through previous work, as well as to the content and layout of information presented in a downloadable generic version of the mHealth app. A skilled facilitator (DEK) led the meetings based on a developed facilitation guide. A notetaker (MGD) and an audio recorder documented the meeting proceedings.

### Recruitment

Unlike study participants, patients’ and caregivers’ role in patient engagement activities was to represent lived experiences rather than be representative of them [21]. Thus, given the focus of the mHealth app under development, advisory panel membership was based upon the shared experience of having undergone or cared for someone who had undergone cardiac surgery at our study hospital. Specifically, patients who underwent the cardiac surgery procedure within the previous 2 years (2017-2018) at the study hospital and consented to be listed in a database of individuals interested in participating in future research and their caregivers were approached for advisory panel membership. As women are underrepresented in cardiac research and to obtain perspectives that spanned the gamut of cardiac surgery procedures most typically carried out at our study hospital, panel members were selectively chosen for diversity in sex and procedure type. Individuals were excluded if they could not read or communicate in English. Recruitment was targeted at 10-12 individuals based on our and others’ experiences with group dynamics and group size. For example, advisory panels within Patient-Centered Outcomes Research Institute range between 10 and 24 members [22], whereas group sizes of 9-12 and 6-12 are commonly recommended for group processes focused on idea generation and discussion, such as the nominal group technique [23] and focus groups [24], respectively. Smaller group sizes (n=4-12) are large enough to facilitate discussion while leaving room for balanced participation [25].

### Impact of Patient Engagement on mHealth App Development

A qualitative exploration was undertaken to determine the impact of patient engagement on the development of the mHealth app. This included description of (1) the key messages generated by the advisory panel, (2) how key messages were incorporated into the development of the mHealth app, and (3) feedback from the developers of the mHealth app about the key messages generated by the advisory panel.

### Analysis

Discussions that occur as part of patient engagement activities do not typically produce data that are thematically analyzed [25], as the purpose of patient engagement is to learn from patient experiences and not interpret patient experiences through the researcher’s lens. Thus, “real-time processing” of information takes place during discussions, and the information that is gathered is generally presented as a list of stakeholder-made recommendations used to support project decision making [25]. Accordingly, the meeting facilitator (DEK) employed common techniques (eg, summarization, reflection, asking clarifying questions) to identify advisory panel members’ key messages during discussions. Two study team members (DEK and AMC) reviewed the research assistant’s notes from all 3 meetings along with transcripts from the second meeting to generate a list of key messages about the design and content of the mHealth app. These key messages were presented by a study team member (DEK) to the developers of the mHealth platform to guide the design and content of the mHealth app. In addition, advisory panel members’ sociodemographic characteristics, as obtained from our database of individuals interested in participating in future research (patients) and self-report (caregivers), were summarized with medians (25th and 75th percentiles) or counts (percentages). These descriptive statistics were calculated using Stata version 13.0 (Stata Corp).

## Results

### Sociodemographic Characteristics of the Participants

Ten individuals (6 patients and 4 caregivers) participated in the advisory panel. The select sociodemographic characteristics of the advisory panel members are shown in Table 1. Each caregiver (n=4) was a patient’s (n=4) spouse. Two of the patients did not have a caregiver attend any of the advisory panel sessions.

**Table 1:** Select sociodemographic characteristics of the advisory panel members (N=10).

Variable			Patients (n=6)		Caregivers (n=4)
Age (years), median (IQR)			74 (72-76)		N/A^a^
Females, n (%)			3 (50)		3 (75)
**Ethnicity, n (%)**					
	White/Caucasian/European	5 (83)		4 (100)	
	First Nations/Inuit/Metis	1 (17)		0 (0)	
**Procedure type, n (%)**					
	Aortic valve replacement	3 (50)		N/A	
	Aortic valve replacement/coronary artery bypass grafting	1 (17)		N/A	
	Aortic valve replacement/mitral valve replacement	1 (17)		N/A	
	Mitral valve replacement	1 (17)		N/A	

^a^N/A: not applicable.

### Key Messages About the Design of the mHealth App

A summary of the advisory panel members’ key messages about the design and content of the mHealth app is shown in Table 2. Key messages were about the design features of the mHealth app related to access, tracking, synchronization, and reminders. Specific key messages about the mHealth app design are shown in Table 2.

**Table 2:** Key messages about the design of the mobile health app.

Key messages (the design of the app should include the ability to…)	Overarching message category
Access information ahead of medical appointments	Access
Access information offline	Access
Share access to the mobile health app with caregivers and family	Access
Track prescribed medications and exercises that are assigned both in hospital and during outpatient rehabilitation	Tracking
Synchronize information from medical devices	Synchronization
Schedule reminders to take medications	Reminders
Provide daily reminders about assigned exercises and general physical activity recommendations	Reminders

### Key Messages About the Content of the mHealth App

During discussions of the study’s 2 open-ended questions and the generic version of the mHealth app, content-specific messages centered around medical terms, professional roles, information specific to cardiac surgery procedures and recovery, educational videos, and travel before/after surgery. When discussing the study’s predefined categories of information, key content-specific messages about (1) nutrition related to what to eat, (2) medications, including drug interactions, (3) resources, including medical devices, and (4) physical activity related to addressing fears, as well as providing information, recommendations, and instructions were generated by the advisory panel. Specific key messages about the mHealth app content are shown in Table 3.

**Table 3:** Key messages about the content of the mobile health app.

Key messages (the app’s content should include…)	Overarching message category
Definitions of key terms	Medical terms
Cardiac surgery team contact information	Professional roles
Information about the functions of the different operating room personnel	Professional roles
Information specific to the different cardiac surgery procedures	Cardiac surgery procedures
Information about postoperative recovery, including why you might have a chest tube	Cardiac surgery recovery
Videos that explain the different cardiac surgery procedures	Educational videos
Information about driving/traveling after cardiac surgery	Travel
Instructions on what to eat during the perioperative period	Nutrition
Recipes geared toward those who are looking to adopt a more heart-healthy lifestyle	Nutrition
Potential drug interactions	Medications
Resources for medical devices	Resources
Information that helps address fears around engaging in physical activity before and after cardiac surgery	Physical activity
Information about and instructions on the types of physical activities patients can and cannot engage in (specific to procedure and perioperative period)	Physical activity
Instructions on the physical activity and specific exercises a patient should do if they miss a cardiac rehabilitation session	Physical activity
Instructions on how to complete exercises assigned both in hospital and during outpatient rehabilitation	Physical activity
General physical activity recommendations	Physical activity

### Incorporation of the Key Messages Into the Development of the mHealth App

Key messages about the design and content of the mHealth app were compiled and sent to the mHealth app developers by the study coordinator (DEK). These were then directly incorporated into the mHealth app as long as they could be supported by the existing functionalities of the underlying platform. For example, the platform did not support the scheduling of reminders by users, identifying drug interactions, or synchronizing with other devices. Verbal and written feedback from the mHealth app developers indicated that the key messages were a richer source of information and provided more guidance than typically received from past clients. In particular, the mHealth app developers noted that key messages resulted in the integration of a vast range and volume of information and resources, instead of ones primarily focused on surgical information, content geared toward expectations management, and an expanded focus of the mHealth app to include caregivers and other family so that these stakeholders may be directly included in the provision of information, allowing them to be better informed, prepare along with the patient, and be involved in recovery planning.

## Discussion

### Principal Findings

Our findings demonstrate that engaging patients and caregivers in research through the formation of an advisory panel yields a rich source of usable information to guide the development of an mHealth app for the perioperative period of cardiac surgery. Advisory panel members generated 7 key design-specific messages centered around access, tracking, synchronization, and reminders, as well as 16 key content-specific messages centered around medical terms, professional roles, cardiac surgery procedures and recovery, educational videos, travel, nutrition, medications, resources, and physical activity. These findings are novel because despite the increased recognition of the importance of involving patients in research, patient engagement remains underutilized in many health research areas, including mHealth design [9] and cardiac surgery. Further, while patient input is more regularly sought in the commercial technology arena, it is often obtained through focus groups or pilot testing aimed at gathering proprietary data; it is rare that patients and caregivers are engaged as partners and cocreators of mHealth.

Several characteristics of our patient engagement activities likely contributed to the gathering of useful information. The first is the deliberate intention to create an environment that supported patients’ and caregivers’ integration into research through activities that targeted the guiding principles that underlie patient engagement [11] and as led by a skilled facilitator. Second, a mixture of broad and focused open-ended questions was used to gather spontaneous feedback as well as feedback related to categories of information based on our previous work. Interestingly, during discussions of the broad, open-ended questions, topics raised tended to concern the potential benefits of the mHealth app. For example, some of the topics raised by the panel included the technology’s potential to change how patients and caregivers interact with information to better support patient engagement with their health care plan (eg, through the ability to access information ahead of an appointment to prepare questions or know what to expect, by allowing them to fact-check what they thought they heard during appointments without having to rely on outside sources like internet searches) and the potential for caregivers to become more involved in the patient’s journey. Discussions of more focused questions produced key messages more directly related to the design and content of the mHealth app. Third, advisory panel members were selected based on whether they had undergone cardiac surgery within the past 2 years, thereby ensuring accurate recall of their experience and elaborating on the information they did and did not receive as part of their patient-provider interaction. This would have had a positive impact on their abilities to contribute to conversations. Fourth, the advisory panel met on multiple instances, which allowed advisory panel members to reflect on the study questions and their experiences alone or with caregivers and other individuals who supported them during their patient journeys and then to bring these reflections back to enrich discussions in subsequent meetings. Finally, the advisory panel included both patients and their caregivers, which provided a breadth of experiences, and turned out to be timely, given the patients’ statements on the potential of the mHealth app to allow caregivers to be more involved in the patient’s journey.

With the increase of older adults being offered cardiac surgery, there is an urgent need to provide a high level of patient-centered value and quality in our perioperative management. The use of evidence-based ERPs has resulted in more rapid and optimal recovery than that with traditional perioperative methods (ie, improved survivorship) in patients who have undergone cardiac surgery [26]. Although published guidelines provide an important framework from which to develop clinical pathways [27], implementation remains challenging, and therefore, the protocols are underutilized. It is anticipated that the approach of involving patients and caregivers in the development stage will enable the health care team to focus on patient-caregiver value in the subsequent implementation phase that will ideally translate to a sustainable process. To this end, the findings from this study have provided a deeper understanding of patient and caregiver needs pertaining to information delivery about various aspects of perioperative care and the potential role of mHealth in supporting these recommendations.

### Limitations

This study has some limitations that warrant mention. Logistical constraints shaped our patient engagement approach. For example, while we engaged patients and caregivers at specific time points within the study, we did not continually involve them throughout the project as full research coinvestigators. Had there been continual engagement, there would have been other points of input and the nature of advisory panel members’ relations with the study would have been different. That said, it is important to note that advisory panel members were invited to be coauthors on this manuscript, both to further support the establishment of authentic research partnerships and to ensure that the manuscript accurately reflects their voices and ideas. We also plan to engage advisory panel members further in the reevaluation and revision of the mHealth app prior to its adoption as a standard of care tool to be used within the Cardiac Sciences Program at St. Boniface Hospital.

### Conclusions

In an era of increasingly utilized mHealth technologies for optimizing health care delivery, we demonstrated that patient engagement may successfully facilitate the development of an mHealth app whose design and content are driven by the lived experiences of patients who have undergone cardiac surgery and their caregivers. The result was a detail-oriented and patient-centered mHealth app that helps to empower and inform patients and their caregivers across the perioperative period of cardiac surgery. Applications of different patient engagement approaches and their effects on mHealth app development, measures of feasibility, and health outcomes warrant further study.

## Appendix

Multimedia Appendix 1 An overview of our study's approach to creating an environment conducive to patient engagement.

## Abbreviations

ERP: enhanced recovery protocol
mHealth: mobile health
