# Supplementary material for: Patient Engagement in the Design of a Mobile Health App That Supports Enhanced Recovery Protocols for Cardiac Surgery: Development Study
Source: JMIR Perioper Med. 2021 Nov 30;4(2):e26597. doi: 10.2196/26597 (PMC8672287; doi:10.2196/26597)
Supplement: Multimedia Appendix 1 [file periop_v4i2e26597_app1.pdf]

Multimedia Appendix 1: Cocreating an environment conducive to patient engagement

Please note, this is a Multimedia Appendix to Chudyk AM, Ragheb S, Kent D, Duhamel TA, Hyra C, Dave MG, Arora RC, Schultz ASH. Patient Engagement in the Design of a Mobile Health App That Supports Enhanced Recovery Protocols for Cardiac Surgery: Development Study. JMIR Perioper Med 2021;4(2):e26597.

Canadian Institutes of Health Research's Patient Engagement Framework includes four guiding principles (ie, mutual respect, inclusiveness, cobuilding, and support) for researchers to adhere to when engaging patients in research [11]. Thus, when developing the engagement plan, our key considerations centered around how to obtain input on the design and content of the mobile health (mHealth) app, as well as embodiment of these four guiding principles in our engagement approach. A description of how these guiding principles are interwoven into this study's engagement activities follows.

*Mutual respect***:** An environment in which advisory panel members and other members of the research team acknowledged and valued each other's expertise and experiential knowledge [11] was fostered through the establishment of a mutual understanding of roles and responsibilities, as well as the importance of experiential knowledge to the study. This included discussing with advisory panel members the roles of the different individuals involved in the study as well as explicitly stating that the role of "academic" researchers was to listen and learn from the lived experiences of advisory panel members.

*Inclusiveness***:** This guiding principle refers to research that integrates a diversity of perspectives as well as that is reflective of patient contributions [11]. Advisory panel members were selectively chosen to be heterogeneous in sex and procedure type to obtain a diversity of perspectives. Strategies to ensure that the research was reflective of patient contributions included employing common techniques (eg, summarization, reflection, asking clarifying questions) to identify advisory panel members' key messages during discussions and bringing all of the advisory panel members' key messages to the mHealth app developers.

*Cobuilding***:** This guiding principle states that patients and other researchers should work together to identify problems/gaps, set priorities, and develop/implement solutions.[11] Although advisory panel members were not involved across the study's research cycle, attempts to incorporate this guiding principle included asking advisory panel members to reflect on the information that they wish they had known and that was important to them during the perioperative period in cardiac surgery (ie, identify problems/gaps), as well as provide input on delivery of this information within the mHealth app (ie, provide solutions through the design and content of the mHealth app).

*Support***:** Main approaches to supporting advisory panel members in contributing fully to discussions and decisions include (a) cocreation of a "safe" environment and offering (b) educational and (c) financial supports [11]. First, for genuine dialogue to occur, individuals should feel safe to share their thoughts and ideas. Since these feelings of safety may look like different things to different people, at the outset of the first meeting, ground rules for interpersonal interactions were cocreated by the group and revisited at the start of subsequent

meetings. A synopsis of these ground rules is presented in Table S1. Icebreaker activities also targeted the creation of a safe space. For example, to help facilitate openness to others' viewpoints, the group engaged in an icebreaker activity called clotheslines or kitestrings [18]. The activity asks all participants to choose between the same sets of contrasting objects and then to explain the reasoning behind their choices. Through sharing perspectives behind decisions that have no right or wrong answers, advisory panel members were encouraged to remember that many different viewpoints can underlie a decision and to be open to opinions that may be different from their own.

Table S1. An overview of meeting ground rules[a]

Ground rule	Brief explanation
E-manners	Silence your phones, put away your emails.
Everyone speaks	To maximize our time together, be sure to speak up if you do not understand something, think we are wasting time, or think we are speeding through an issue that deserves more time.
One conversation	Avoid side conversations and speaking at the same time.
Take a stand	To keep things moving, you may sometimes be asked to choose a single preferred answer or to tell us what you think will work if we identify something that does not work.
Questions first	If you disagree with someone, ask them questions about why they think something works, rather than telling them they are wrong.

ELMO[b]	We have limited time together, so if you think we are getting off-track feel free to state “ELMO” and if the majority of the group agrees then we will move on to another topic.
General roles	The role of advisory panel members is to actively participate and share their knowledge and ideas. The role of researchers is to guide the meetings, create a safe space where everyone feels heard and welcome, and to learn from the lived experiences of advisory panel members.
Start and end on time	Out of respect for everyone, let’s start and end on time.
Parking ideas	To maximize our time together, we will sometimes have to park conversations and/or ideas for future discussion.

[a]Adapted from the work of [19].

[b]Enough already, let’s move on.

Second, educational supports are important for enhancing individuals’ capacity to contribute fully to discussions. Thus, before engaging in discussions about the mHealth app, advisory panel members were provided with the background work and research that informed the current study and received educational supports during meetings, such as a generic version of the mHealth app that was the starting point for the mHealth technology under development and “homework” that aimed to help them prepare for subsequent discussions. The meeting facilitator (DEK) also encouraged advisory panel members to ask any questions they needed answered to be able to provide meaningful input into discussions and a content expert in cardiac surgery (RCA) attended two of the meetings to help answer questions that arose related to cardiac surgery.

Finally, financial supports should acknowledge the time, knowledge, and expertise that patient partners contribute to a research project, in addition to covering expenses related to the activities they engage in [20]. Thus, the financial support offered to advisory panel members considered the time they spent at meetings, as well as transportation and parking costs.
